# Strategies to improve homology-based repair outcomes following CRISPR-based gene editing in mosquitoes: lessons in how to keep any repair disruptions local

**DOI:** 10.1186/s12985-022-01859-2

**Published:** 2022-07-30

**Authors:** Micaela Finney, Joseph Romanowski, Zach N. Adelman

**Affiliations:** Department of Entomology, Texas A&M University, 329A Minnie Belle Heep Center, 370 Olsen Blvd, College Station, TX 77843 USA

**Keywords:** DNA repair, Homology-dependent repair, CRISPR, Gene editing, Mosquito, Aedes

## Abstract

Programmable gene editing systems such as CRISPR-Cas have made mosquito genome engineering more practical and accessible, catalyzing the development of cutting-edge genetic methods of disease vector control. This progress, however, has been limited by the low efficiency of homology-directed repair (HDR)-based sequence integration at DNA double-strand breaks (DSBs) and a lack of understanding about DSB repair in mosquitoes. Innovative efforts to optimize HDR sequence integration by inhibiting non-homologous end joining or promoting HDR have been performed in mammalian systems, however many of these approaches have not been applied to mosquitoes. Here, we review some of the most relevant steps of DNA DSB repair choice and highlight promising approaches that influence this choice to enhance HDR in the context of mosquito gene editing.

## Introduction

Major advancements in genetics, molecular biology, and vector biology have led to a massive increase of information about mosquitoes that vector critical viral pathogens, including high quality genome assemblies of major arbovirus vectors like *Ae. aegypti* [1] and *Cu. quinquefasciatus* [2]. The increase in available vector information in combination with the inability of existing approaches (insecticides, drugs, vaccines) to control many mosquito-borne viruses, has led to investigations into genetic tools and their potential applications for vector control or transmission reduction. Genetic approaches have yielded transgenic mosquito strains unable to transmit important viral pathogens such as dengue, Zika and chikungunya viruses [3]. Likewise, genetic approaches have allowed the development of transgenic strains that result in the premature death of vector mosquitoes through release of insects with a dominant lethal (RIDL), a modification of the classic Sterile Insect Technique (SIT) [4, 5]. Gene drive approaches have been developed to accelerate the spreading of such engineered genes into mosquito populations [6]. Gene drive refers to a process of biased inheritance, where a genetic element or an allele is increased in frequency across a population, even if its presence causes a fitness cost [7]. Currently, many gene drive designs are based on the Clustered Regularly Interspaced Short Palindromic Repeats (CRISPR)—CRISPR-associated protein 9 (Cas9) system. Jinek et al. [8] revealed site-specific dsDNA cleavage via a family of endonucleases and proposed the use of the system in programmable genome editing. Since then, the CRISPR-Cas9 system has been used across animals, plants, and microorganisms for editing cells, tissues, and whole organisms [9]. Homing-based gene drives which utilize the CRISPR-Cas9 system act in a two-step process: CRISPR-Cas9 creates a double-strand DNA break (DSB) at a pre-selected site allelic to the gene(s) to be driven; with subsequent repair via homology-based processes resulting in a duplication of the starting gene [10].

Site-specific gene editing approaches using tools such as CRISPR-Cas9 also rely on homology-directed repair (HDR) for the initial insertion of genetic material, such as genes that could decrease the competence of mosquitoes to transmit viruses. In this context, DSBs are induced with CRISPR-Cas9 and exogenous donor DNA is provided to serve as a template for ectopic HDR and hence chromosomal integration of a gene (or genes) of interest. Under these conditions, however, ectopic HDR is rare, since this break will occur irrespective of cell cycle stage and relies on the ability of the template DNA donor to be within proximity of the break site for repair, representing a severe bottleneck in the testing/evaluation of novel anti-viral approaches. While homing gene drive systems have been reported with HDR rates of 95% or more [11–13], even very small numbers of mis-repair events could result in complete resistance to the gene drive [14] and thus there remains a strong interest in mechanisms that increase the rate of HDR-based repair for gene drive as well. While the field of mosquito genetics/arbovirology is relatively small, similar problems are faced by the much larger enterprise of gene editing/gene therapy in human medical applications. As in mosquito gene editing, gene therapy applications also rely on inefficient ectopic repair, with even rare mis-repair events posing potential health and safety concerns. In this review, we examine recent strategies and breakthroughs in vertebrate systems related to the development of methods to bias DNA repair outcomes only at the DSB of interest, while leaving global repair alone. We discuss how these efforts to make the local DSB of interest more favorable for HDR with modified nuclease systems, small molecules, and donor DNA complexes could be applied to vector biology and the interruption of virus transmission.

## DSB repair: key players and choices

DSBs are the most cytotoxic form of DNA damage and as a result eukaryotic cells have developed a sophisticated DNA damage response (DDR), consisting of a complex series of cellular signaling networks which handle the detection, cell cycle checkpoints, and ultimately the repair [15]. DSBs can be caused from internal agents such as reactive oxygen species (ROS) or metabolic processes, or by external agents such as ionizing radiation and some chemotherapeutic drugs. Most cell types spend a considerable portion of their life in a resting state and repair of DSBs is critical for genomic stability and mutations which occur in cell cycle-arrested cells [16]. It is estimated up to 10 DSBs occur per cell per day in dividing mammalian cells; these can lead to deletions, chromosome loss, or translocations if not repaired correctly, and can lead to cell death if not repaired at all [15, 17, 18]. The major DSB repair pathways are non-homologous end-joining (NHEJ) and homology-dependent repair (HDR), the latter of which can follow several sub-pathways, including single-strand annealing (SSA), alternative non-homologous end joining (A-NHEJ), synthesis-dependent strand annealing (SDSA), and break-induced replication (BIR). HDR is considered an error-free template-dependent pathway and can result in perfect repair via sequence homology found in a sister chromatid or an exogenously introduced template, such as a PCR amplicon or plasmid [19, 20]. All homology-dependent mechanisms of repair begin with end resection (5’-3’ resection), whereas NHEJ repairs DSBs via blunt end ligation, which does not utilize sequence homology. NHEJ is considered error-prone, often resulting in mutations in the form of small insertions or deletions (indels). This repair pathway can occur during any stage of the cell cycle but is the dominant pathway during G1 when the sister chromatid is not available [18, 21]. Despite the pathway often leading to errors during repair, NHEJ is often preferred over HDR within a cell due to its high-capacity, rapid action and activity throughout the cell cycle [17].

**Fig. 1.**
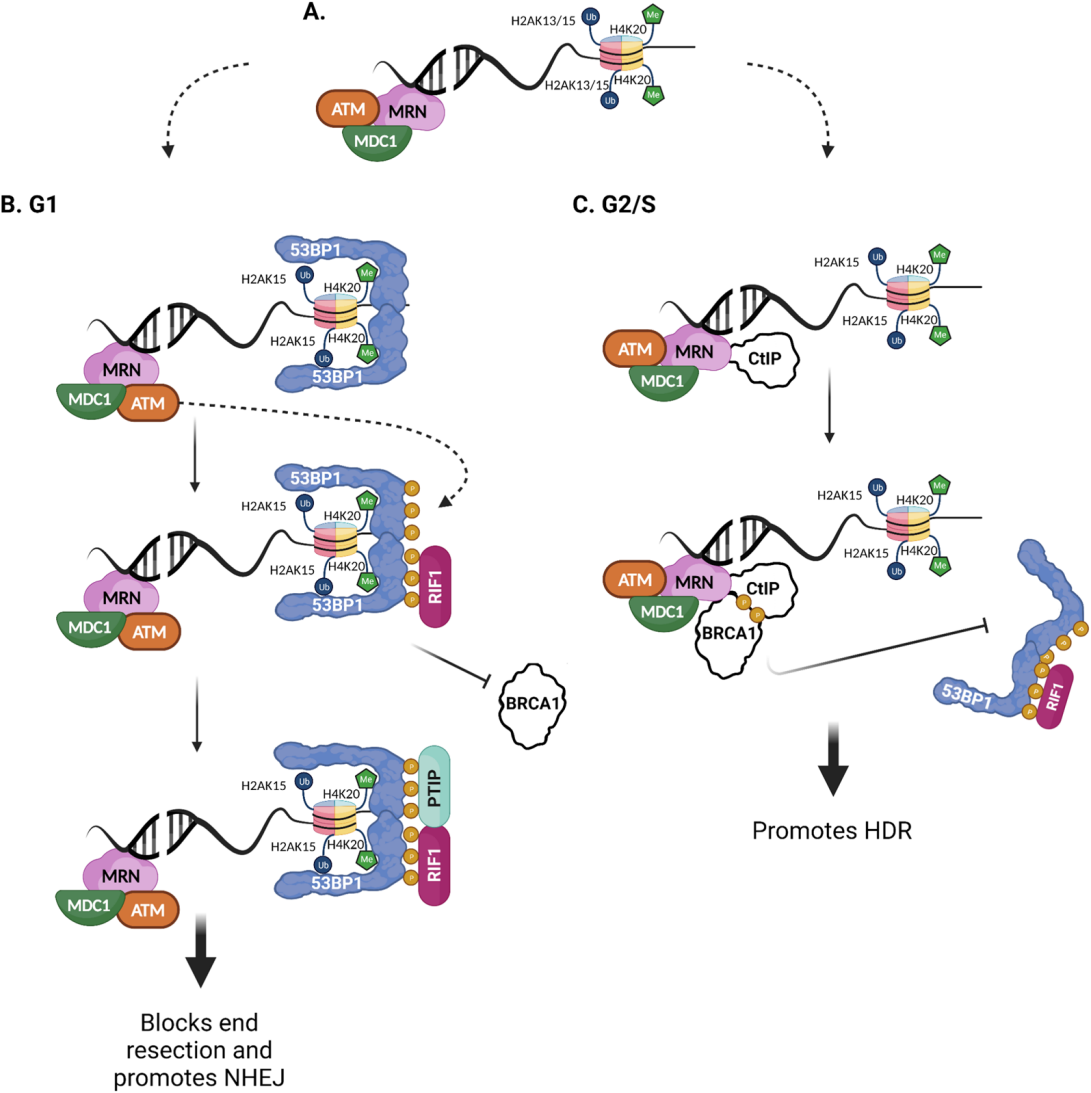
53BP1 influences repair pathway choice. **A** DSB which has had initial repair factors recruited (MRN complex, ATM, MDC1) and adjacent to a nucleosome with ubiquitylated Lys15 of histone 2A (H2AK15ub) and mono- or di-methylated Lys20 of histone 4 (H4K20me1/2). **B** In G1 phase, 53BP1 binds to H2A15ub and H4K20me1/2 via its UDR motif and tudor domain respectively. RIF1 is recruited to 53BP1 via binding to ATM-phosphorylated residues. BRCA1 foci formation is inhibited in G1 via 53BP1 and RIF1, where the N terminus ATM target sites in 53BP1 are necessary for its ability to recruit and interact with RIF1 and PTIP. ATM-phosphorylation of 53BP1 leads to recruitment of other NHEJ-promoting factors such as PTIP and EXPAND1, and leads to blocking of end resection and promotion of NHEJ. **C** In G2/S phases, CtIP is recruited by and bound to the MRN complex. After CtIP is phosphorylated, BRCA1 binds, 53BP1-RIF1 are inhibited from binding to chromatin, and end resection and HDR are promoted. For all panels, colored fill indicates protein factors are conserved in vector mosquiotes, while while fill (CtIP, BRCA1) indicates repairs factors that appear to be absent in mosquitoes

DSB repair requires at least three distinct operations: damage detection, control of cell cycle and transcriptional programs in response to damage, and mechanisms for repair. Both NHEJ and HDR rely on the Mre11-Rad50-Nbs1 (MRN) complex upstream of the ultimate repair pathway choice. The MRN complex functions in all three facets of DSB repair; the complex is a DSB sensor, a co-activator of cell cycle checkpoint signaling, and a DSB repair effector [22]. The MRN complex recruits the ataxia telangiectasia mutated (ATM) kinase to the DSB that in turn sets off a chain of events ultimately leading to the ubiquitination of histones H2A/H2AX [21]. Following ubiquitination, a repair pathway choice is made based on cell-cycle and the subsequent protein recruitment (Fig. 1A). In G1, 53BP1 is recruited, inhibiting end resection and leading to NHEJ (Fig. 1B), while in S/G2, BRCA1 is recruited (binding to CtIP), displacing 53BP1 and stimulating end resection which leads to HDR (Fig. 1C) [23].

A key determinant in DSB repair pathway choice, and the first NHEJ-promoting protein recruited downstream of DSB-recognition by the MRN complex, 53BP1 acts as a positive regulator of NHEJ. Human 53BP1 has specific structural elements: BRCA1 carboxy-terminal (BRCT) repeats, 28 Ser/Thr-Gln (S/T-Q) sites in the amino terminus, and a minimal focus-forming region (FFR) which contains an oligomerization domain (OD), a Glycine and Arginine-rich (GAR) motif, a tandem Tudor motif, and a ubiquitylation-dependent recruitment (UDR) motif [23, 24]. In humans, 53BP1 directly binds to RNF168-ubiquitinylated H2AK15 via its UDR and mono- and dimethylated Lys20 of histone 4 (H4K20me1 and H4K20me2) via its Tudor domain [23]. 53BP1 is phosphorylated by ATM which leads to the recruitment of RIF1 (RAP1-interacting factor 1) and PTIP (Pax2 transactivation domain-interacting protein) effector proteins [23, 25]. The N terminus of human 53BP1 is responsible for the binding of RIF1 and PTIP, while the tandem BRCT domains bind to EXPAND1 and p53. The chromatin bound 53BP1-RIF1 inhibits BRCA1 from associating with MRN and CtIP and protects DSB ends from processing via inhibiting end resection and restricts HDR to S and G2 phases of the cell cycle [26], though the precise mechanism of how end resection is inhibited remains unresolved. 53BP1 is also responsible, in part, for activation of ATM [27].

## Global disruption of NHEJ to promote HDR

Strategies for promoting HDR have been developed based on the overall inhibition of NHEJ repair [17]. Suppression of NHEJ has been achieved via knock-down, knock-out, or chemical inhibition of key NHEJ proteins, such as ligase IV, KU70/KU80, and DNA-PKcs [28–33]. SCR7, a DNA ligase IV inhibitor, acts on the binding domain of lig IV to reduce its affinity for DSBs and inhibits its function [29, 32]. Various human cell lines derived from breast, cervical, lung, and ovarian cancers; and fibrosarcoma displayed a decrease in proliferation upon dose-dependent treatment with SCR7 [32]. In addition, an HR-deficient cell line had increased sensitivity to SCR7 and enhanced cell death compared to its wild-type line. These results show in the absence of HR and inhibition of lig IV, DSBs accumulate and lead to cell death without repair. SCR7 treatment of leukemia cells resulted in PARP1 cleavage and DNA fragmentation, as well as increased cell death. Another strategy involved lig IV knockouts in mice [33], where over 50% of the ligase IV coding sequence was replaced with a neomycin-resistance gene cassette resulting in an embryonic lethal mutation. Although phenotypically similar to their wild-type siblings, intercrossings of the heterozygous (*LigIV*^+/−^) mice led to no adult mice homozygous for the mutation (*LigIV*^−/−^). Other issues caused by the mutation were a defect in B-cell development and marked sensitivity to ionizing radiation and blocked cell growth in *LigIV*^−/−^ mouse fibroblast cells. These results are similar those of studies performed with Ku80- and Ku70-deficient mice, with the exception of only lig IV-deficient mice did not produce viable progeny [34, 35]. Higher frequency or prolonged G2 cell cycle arrest has been demonstrated in Ku70^−/−^ and Ku80^−/−^ mouse embryonic fibroblast (MEF) cells or via DNA-PKcs inactivation in ATM^−/−^ human fibroblasts [36, 37]. Ku70^−/−^ and DNA-PKcs^−/−^ MEF cells also exhibit increased irradiation (IR) sensitivity and reduced cell survival probability compared to their WT MEF cells [38]. When IR was combined with a DNA-PKcs inhibitor, Nu7441, in SUNE-1 cells (derived from a patient with undifferentiated NPC); the number of cells which were arrested in G2/M were significantly higher than IR alone at 24- and 48-h post irradiation [38]. In addition to prolonging cell cycle progression, Nu7441 in combination with IR led to impaired DSB repair and activating cell cycle checkpoints in SUNE-1 cells. Permanently disabling NHEJ factors in mice has also had severe deleterious effects, including bone marrow failure, stem cell aging, and cancer susceptibility [32, 33, 39]. Taken together, strategies aimed at increasing rates of HDR by globally disrupting NHEJ factors (knockout or knockdown) are likely to face difficulties due to high fitness costs associated with losing NHEJ.

### Towards local inhibition of NHEJ

Since 53BP1 binds only ubiquitylated histones, Canny et al. [28] screened potential 53BP1 inhibitors from a library of ubiquitin-variants (Ubvs) which were initially documented to inhibit other ubiquitin-binding proteins. They found 4 distinct Ubvs which bound selectively to the 53BP1 Tudor-UDR region or to a 53BP1 fragment containing the Tudor domain only. UbvG08, which displayed the highest 53BP1 binding via phage ELISA, differed from the WT-Ub at only 7 positions. As 53BP1 binds to dimethylated histone H4 Lys20 (H4K20me2), the affinity of UbvG08 was evaluated against methyl-lysine peptides and found to bind 53BP1 by 2 orders of magnitude tighter. The crystal structure of UbvG08 bound to the Tudor domain of 53BP1 allowed for the identification of residues positioned on the contact surface of UbvG08 which allow for direct interaction of their side chains with 53BP1. Of the seven residue differences between UbvG08 and ubiquitin, four had favorable positioning: L70, L2, and P69 form favorable hydrophobic contacts and K66 forms an electrostatic interaction with 53BP1. Reversion of the seven substitutions in UbvG08 to their WT-Ub counterparts led to reduced or abolished UbvG08-53BP1 binding, with reversion of P69L and L70V displaying the strongest effect on binding. Conversely, mutating the equivalent residues in WT-Ub into their UbvG08 counterparts led to robust 53BP1-binding. To assess inhibition of 53BP1 via intracellular expression of UbvG08, a flag-tagged version of UbvG08 was prepared (i53) in order to disable ubiquitin-dependent interactions within the cell, as well as a UbvG08-deficient mutant (i53-DM) with P69L and L70V mutations reverted to WT.

For experiments in human bone osteosarcoma epithelial (U20S) cells, i53 strongly suppressed 53BP1 recruitment to DSBs and this interaction was specific to 53BP1, as it did not affect γ-H2AX and BRCA1 focus formation. In G1 cells, transfection with i53 induced BRCA1 accumulation at DSB sites at rates similar to 53BP1-knockout cells. Across nine immunoprecipitation–mass spectrometry experiments, the only protein which interacted with i53 in two or more was 53BP1, demonstrating that i53 is a selective binder of 53BP1 and inhibits not only the recruitment, but also the function of 53BP1. AAV-delivered i53 showed stimulation of HDR in human and mouse cells. While promising, expression of i53 alone disrupts 53bp1-dependent end-joining at all DSB sites across the genome. However, a potential fusion of i53 to Cas9 protein could allow more localized control over repair outcomes.

## Modified nuclease systems to bias for HDR only at targeted DSBs

Previously, Sternberg et al. [40] reported how PAM recognition allows Cas9 to identify potential target sites to generate DSBs. Upon investigation of Cas9:RNA binding kinetics to DNA, DNA curtain assays used to test protein-nucleic acid interactions revealed that Cas9 remains tightly bound to DSB ends after the DSB is induced. Since this discovery, various enzymes have been linked to Cas9 in order to influence DSB repair pathway choice for desired gene editing outcomes. To date, these modifications have been able to increase rates of HDR by restricting end-joining, by promoting strand invasion/ DSB end resection, or through the spatial and/or temporal control of Cas9 activity itself.

### Local inhibition of NHEJ through dominant negative 53bp1

Jayavaradhan et al. [24] pursued local NHEJ inhibition via the use of a potential dominant negative (DN) version of 53BP1 fused to Cas9 in HeLa cells, capable of binding to damaged chromatin but incapable of inhibiting end resection or recruiting end-joining factors. The 5 DNs (DN1, DN1S, DN2, DN3, and DN4) designed included various combinations of regions of the FFR of 53BP1, with all DN including at least the Tudor domain which is responsible for 53BP1-H4K20me2 interaction. The regions of 53BP1 which bind RIF1, PTIP, and EXPAND1 (the N terminus, as well as the tandem BRCT domains), were not included in the DN fragments in order to eliminate recruitment of downstream NHEJ proteins. In HeLa cells, DN1 and DN1S co-localized with 53BP1 at irradiation induced foci (IRIF). When expressed at higher levels, DN1S completely inhibited localization of 53BP1 to IRIF, with DN1S IRIF observable but no IRIF present with only endogenous 53BP1 bound. Similar to UvsG08, DN1S expression led to a significant increase of recruitment of BRCA1 to DSBs as compared to empty vector transduced cells. A large decrease of RIF1 recruitment was observed in cells expressing DN1 or DN1S, as well as an increase of BRCA1 foci in S/G2 phase. Most notably, in G0/G1 phases, where NHEJ is normally promoted over HDR in G1, a significant increase in tBRCA1 foci was observed without affecting the cell cycle.

To verify if Cas9-DN1S exhibited global or local NHEJ inhibition, experiments were performed with an NHEJ-reporter HeLa cell line, in which excision of the puromycin-resistance gene with flanking I-SceI recognition sites via I-SceI and repair by NHEJ results in GFP expression. Cells were transduced with DN1S or Cas9-DN1S and transfected with the I-SceI plasmid. Resulting expression of GFP in cells was similar between Cas9-DN1S and empty vector transduced cells, while DN1S cells displayed a significant reduction in GFP expression. These results suggest CRISPR/Cas9 fusion to DN1S confers specificity, while DN1S alone affects cellular NHEJ globally. Across different loci and cell types, Cas9-DN1S fusion nucleases led to a two–threefold increase in HDR and a three–fourfold reduction in NHEJ locally. Use of a dominant negative form of 53BP1 fused to Cas9 can thus significantly reduce the amount of NHEJ repair at a nuclease cleavage site, without introducing the negative effects of global NHEJ inhibition [24, 28].

In *Ae. aegypti*, there remains a substantial knowledge gap in regard to the functions carried out by key HDR factors such as BRCA1 and RAD52, which appear to be absent [21, 23]. Mota et al. [21] also did not identify a 53BP1 ortholog in *Ae. aegypti*, however this may be due to an overly stringent similarity cutoff, as there is one protein [41] which appears to contain both the p53-binding protein-1 Tudor domain (Interpro ID: IPR015125) and BRCT domain superfamily (Interpro ID: IPR036420), making this gene a strong candidate for consideration as the *Ae. aegypti* 53BP1 ortholog. As both RIF1 and PTIP are also conserved in mosquitoes, it appears possible that 53BP1 plays a similar role in promoting NHEJ/restricting resection, though this requires experimental confirmation. As 53BP1 is the first NHEJ promoting protein recruited to a DSB and blocks end resection, it may be an ideal target for local inhibition of NHEJ [42].

### Fusions of targeted nucleases with Pro-HDR factors

**Fig. 2:**
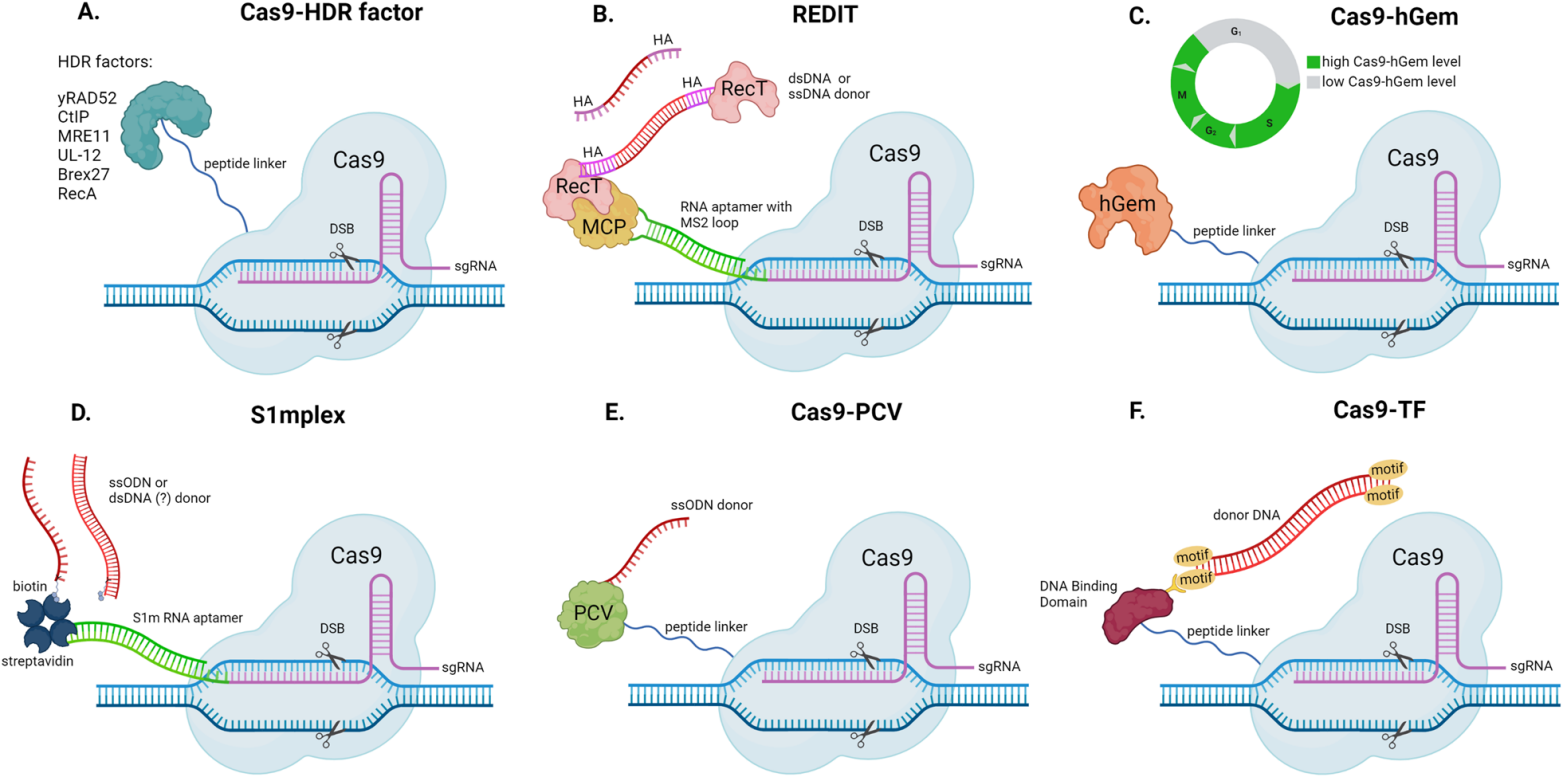
Modified nuclease systems to bias HDR at specific DSBs. **A** Schematic of HDR factors tethered to Cas9 via peptide linker can promote strand-invasion (Cas9-yRAD2, Cas9-Brex27), end-resection (Cas9-CtIP, Cas9-MRE11, Cas9-UL12), or single-strand annealing (Cas9-RecA) at a DNA double-strand break. **B** Diagram of the REDIT system composed of Cas9, sgRNA, RNA aptamer with MS2 loop, MS2 coat protein (MCP), RecT, and either single-strand DNA (ssDNA) or double-stranded DNA (dsDNA) donors. **C** Visual representation of Cas9-hGem levels during cell cycle stages and Cas9-hGem construct. **D** Overview of S1mplex components: Cas9, sgRNA, RNA aptamer, streptavidin, biotin, and either ssDNA or dsDNA donors. **E** Cas9 tethered to PCV, an HUH endonuclease forming a covalent bond with ssODN donor. **F** A transcription factor DNA binding domain fused to Cas9 with a peptide linker binding to motifs presents donor DNA

As opposed to limiting NHEJ, an alternative would be to directly promote HDR at specified DSB sites through the linkage of Cas9 to pro-HDR factors that can promote strand invasion or recombination of donor DNA at the DSB site. In yeast, the repair protein RAD52 (yRAD52) can facilitate strand invasion of replication protein A (RPA)-coated ssDNA in the presence of RAD51, however the human RAD52 (hRAD52) cannot [43]. Shao et al. expressed a Cas9-yRAD52 fusion construct (Fig. 2A) in human HEK293T cells and porcine PK15 cells and assessed its ability to promote ectopic HDR using different donor DNA types (plasmid, PCR product, ssDNA). Compared to cells transfected with a plasmid containing wild type Cas9, cells transfected with yRAD52 or the Cas9-yRAD52 fusion construct exhibited increases in HDR frequency independent of donor DNA type [44]. The Cas9-yRAD52 fusion construct was also applied to chicken cells and reported a threefold increase in HDR compared to wild type Cas9 when single-stranded oligodeoxynucleotides (ssODNs) were used as donor DNA [45]. RAD52 is absent in mosquitoes, however since yRAD52 interacts with RAD51, which is conserved in mosquitoes, it is possible that application of a Cas9-yRAD52 can increase rates of HDR in a similar manner.

As HDR depends completely upon resection of the DSB ends in a 5’-3’ manner to expose ssDNA overhangs that enable searches for homologous sequences [46–48], nuclease fusion constructs that promote end resection at Cas9-induced DSBs have been reported to enhance ectopic HDR [49–51]. One key resection protein in mammals is CtIP. Fusion of the N-terminal domain of CtIP to Cas9 (Fig. 2A) increased rates of ectopic HDR by twofold or more at different loci compared to a wild type Cas9 in human cell lines, human iPS cells, and rat zygotes [49]. One limitation of this approach, however, is this improvement in HDR frequency was found to be dependent on the gRNA used and thus requires testing of multiple gRNAs. While CtIP has not been identified yet in mosquitoes [21], other DNA damage response factors that interact with CtIP such as the MRN complex, ATM, and cyclin dependent kinase (CDK) are present, suggesting that if used in mosquitoes, hCtIP may exhibit similar catalytic activity [20, 21, 41, 52]. Similar to CtIP, fusion of Cas9 to MRE11 (Fig. 2A) has also been shown to increase ectopic HDR in human cells up to twofold [50]. A portion of the UL12 alkaline exonuclease derived from herpes simplex virus type 1 has also been shown to recruit the cell’s endogenous MRN complex to DSB sites. Fusion of Cas9 to a 126 amino acid portion of the UL12 N-terminal domain (Fig. 2A) reported a twofold increase in ectopic HDR when compared to wild-type Cas9 [51].

During HDR, RAD51 is deposited by BRCA2 on resected ssDNA overhangs to form RAD51/ssDNA nucleoprotein filaments [53, 54]. Previous studies have identified a 36 amino acid motif encoded by BRCA2 Exon 27 (Brex27) that binds RAD51 to stabilize the RAD51/ssDNA nucleoprotein filaments [55]. Due to its ability to interact with RAD51, fusion of the Brex27 motif to Cas9 was hypothesized to increase HDR and strand invasion compared to wild type Cas9 (Fig. 2A). Knock-in rates of a plasmid-derived GFP donor sequence into the *AAVS1* locus in human cell lines was 2.5-fold greater with a Cas9-Brex27 fusion (miCas9), compared to those transfected with wild type Cas9 and the GFP donor [56]. Increasing the GFP donor sequence from 1 to 3 kb reduced overall HDR efficiency, however a two–threefold increase in HDR was still observed when comparing miCas9 to wild type Cas9 [56].

RecT is a roughly 270aa long single-stranded DNA annealing protein (SSAP) derived *E. coli* and is similar to the bacteriophage lambda bet (Redβ), and is capable of multi-kilobase recombineering in microbial systems [57–60]. RecT is able to bind to either ssDNA or dsDNA and promote strand exchange of homologous sequences. A Cas9-RecT fusion, RecT Editor via Designer-Cas9-Initiated Targeting (REDIT), was constructed to increase the rate of HDR and improve integration efficiency of larger transgenes through HDR (Fig. 2B). In this system, Cas9 was linked to an RNA aptamer containing MS2 stem-loops capable of binding an MS2 coat protein (MCP)-RecT fusion. Compared to wild type Cas9, REDIT was able to increase HDR threefold, as determined by the percent mKate + cells by flow cytometry, following introduction of a 1 kb donor plasmid [61]. While successful, the requirement for RNA aptamer-MCP binding or MCP-RecT introduces additional complications that could reduce overall efficiency. Although the particular mechanism of interaction between REDIT and endogenous HDR repair is not yet confirmed, nor has it been applied to mosquito systems, SSAPs like RecT have been shown to be functionally diverse with specific activities varying depending on the organism they are applied to [62–64]. As a result, it is possible that optimization of REDIT in mosquito systems may require testing of other types of SSAPs in addition to RecT, such as *Escherichia phage lambda* Bet (LBet) or *Bacteriophage T7* gp2.5 (gp2.5), both of which exhibited some success in promoting HDR as well [61].

In contrast to sequence insertion through ectopic HDR, genes or gene segments can be deleted through the single-strand annealing (SSA) pathway, a process that can allow genome engineers to delete segments of DNA that lie in between repetitive sequences [21, 65, 66]. Lin et al. [66] fused Cas9 to the *E. coli* RecA protein (Fig. 2A) and tested its ability to influence DSB repair compared to wild type Cas9 in HEK293T cells using a dual fluorescent reporter system and flow cytometry that measured AsRED and EGFP fluorescence. This Cas9-RecA fusion construct increased SSA by 2.5-fold in HEK293T cells compared to wild type Cas9 [66]. Importantly, this fusion also resulted in a 33% decrease in ectopic HDR, suggesting Cas9-RecA-induced DSBs are preferentially repaired by SSA. In theory, this chimeric Cas9 system could target a transgene such as a gene drive cassette lying in between repetitive sequences to trigger total transgene elimination [67]. Together, these studies suggest the preferential recruitment of HDR factors to a specific DSB site through tethering to Cas9 can result in local shifts in repair outcomes, without the substantial fitness costs of disrupting DSB repair across the genome.

### Use of small molecules to recruit HDR factors

In addition to modified nucleases that can increase rates of HDR, the application of small molecules has been shown to activate pro-HDR factors and increase the efficiency of sequence insertion. RAD51, among other strand exchange repair proteins in the RecA family of recombinases, plays an important role in HDR by replacing replication protein A (RPA) on ssDNA and identifying homologous dsDNA templates that are crucial for strand invasion and DNA synthesis [68, 69]. In a 10,000-compound library screen, Jayathilaka et al. [70] identified the small molecule RS-1 which stimulated human RAD51 (hRAD51)-mediated D-loop activity by 5- to 11-fold by promoting the formation of active presynaptic filaments, depending on the condition. Transfection of a plasmid containing Cas9 and sgRNA paired with chemical treatment of HEK293A cells with RS-1 increased rates of ectopic HDR by three to sixfold using a donor plasmid [69, 71]. While RS-1 has not yet been applied to mosquitoes, RAD51 is an extremely conserved DSB repair protein present in *Aedes, Anopheles,* and *Culex* mosquitoes [41]. Additionally, a chemical screen of roughly 4000 small molecules performed by Yu et al. [72] identified two compounds that increased ectopic HDR across various mouse and human cell types. These small molecules, namely L755507, a β3-adrenergic receptor agonist, and Brefeldin A, an inhibitor of intracellular protein transport from the endoplasmic reticulum to the Golgi apparatus, increased HDR three- and twofold, respectively, when compared to DMSO-treated control cells [72]. Exactly how the use of these two small molecules were able to increase rates of HDR remains unclear, however their ability to increase efficiency of sequence insertion was common in both transformed and primary cells. Importantly, L755507 and Brefeldin A have not been applied to mosquitoes and, as a result, their ability to impact HDR in these disease vectors remains unknown. While small molecule inhibitors would be expected to act across all DSBs in a genome, their action would be extremely limited in time based on dose/half life.

## Spatial and temporal control of DSB induction

One limiting characteristic of HDR is that it occurs almost exclusively in the S and G2 stages of the cell cycle when endogenous donor DNA provided by sister chromatids is available after DNA replication [73–75]. To overcome this limitation, synchronization of Cas9 activity with the S and G2 cell cycle stages has been performed using inhibitory chemicals, namely lovastatin, mimosine, aphidicolin, thymidine, hydroxyurea, and nocodazole. Synchronization of the cell cycle in HEK293T (human primary neonatal fibroblast) and H9 (human embryonic stem) cells via reversible chemical inhibitors and timed delivery of Cas9 ribonucleoprotein (RNP) complexes led to an increase in HDR events at Cas9 cleavage sites when compared to unsynchronized cells [26]. The rate of HDR in unsynchronized cells was 9%, with 31% as the highest frequency of HDR achieved via the chemical inhibitor nocodazole. Once nocodazole released the cells from synchronization, the cells reverted to cycling similar to the unsynchronized cells. RNP transfection data have shown higher cell viability over DNA transfections and this approach of Cas9 RNP-mediated editing resulted in reduced off-target effects, with high levels of on-target editing [26, 76, 77]. Chemical treatment of HEK293T cells resulted in a maximum HDR/NHEJ ratio of 33% at various loci using ssODN as donor DNA [26]. In a similar vein, fusion of Cas9 to the first 110 amino acids of the human Geminin (hGem) protein (Fig. 2C), a transcription factor exclusively expressed during the late S, G2, and M stages of the cell cycle, has been shown to confer nuclear localization of Cas9-hGem and subsequent degradation by the E3 Ubiquitin Ligase complex APC/Cdh1 during G1 [78, 79]. Cas9-hGem(1/110) increased ectopic HDR by 87% when compared to wild type Cas9 in HEK293T cells [79]. Of significance, Geminin is a conserved protein that is present in *Aedes*, *Anopheles*, and *Culex* mosquitoes [41], however Cas9-Gem has not yet been applied to mosquito systems. Additionally, Cas9-Gem has been combined with the REDIT system described above, as was Cas9-CtIP(HE) and the G2/M-arresting arresting chemical Nocodazole. Compared to these systems without REDIT, the Cas9-HE + REDIT, Cas9-Gem + REDIT, and Nocodazole + REDIT increased HDR significantly with rates increasing from roughly three–fivefold [61].

## Donor DNA-nuclease complexes to promote HDR

The presence and proximity of homologous DNA is a major determining factor in DSB repair outcomes and the introduction of homologous template as donor DNA upon DSB induction can increase rates of HDR [80–82]. For this reason, ‘all in one’ systems composed of Cas9, sgRNA, and donor DNA in a single complex have been designed to provide homologous DNA at a DSB immediately upon induction to promote HDR [83, 84]. One of these ‘all in one’ systems, called S1mplex, consists of the Cas9-sgRNA ribonucleoprotein (RNP), a 60 nucleotide S1m RNA aptamer attached to the sgRNA, streptavidin and biotin linkers (Fig. 2D). When S1mplex was paired with a donor ssODN, it increased the ratio of HDR:NHEJ from 2.7-fold to 18.4-fold depending on cell type when compared to the application of its unlinked components [83]. A limitation of S1mplex is it requires additional steps relating to purification of streptavidin and RNA aptamer synthesis to modify the donor DNA and additional recombinant proteins. To overcome this obstacle, Aird et al. [84] designed a system leveraging the DNA binding property of the HUH endonuclease PCV and its ability to form phosphotyrosine covalent bonds with ssDNA (Fig. 2E). Covalent bonding of an unmodified ssODN to the PCV HUH endonuclease-Cas9 fusion construct resulted in up to a 30-fold enhancement of HDR, measured by restoration of mCherry expression via frameshift correction in multiple cell types and target loci. This construct also reported a 20- to 100-fold increase in HDR compared to S1mplex, even with low RNP concentrations [84]. While promising in the context of correcting frameshift mutations or inserting shorter sequences, a limitation of the ‘all in one’ systems is they have only been shown to be effective with smaller ssDNA donor templates. Similarly, fusion of Cas9 to a transcription factor (THAP11) increased rates of HDR two to threefold when the corresponding THAP11 binding motifs were included in donor DNA [85], suggesting proximity of donor DNA at the time of DSB induction can positively influence repair choices in favor of HDR (Fig. 2F).

## Cas9-transposase constructs as an alternative to site-specific transgene integration

Moving away from HDR entirely, site-specific genomic insertion of exogenous DNA can also be achieved via application of Cas9-transposase fusion constructs [86, 87]. This concept combines the site-specificity of RNP complexes with the DNA insertion capability of transposases to deliver and integrate donor DNA at precise genomic locations. These Cas9-transposase complexes use either a catalytically dead form of Cas9 (dCas9) to inhibit DSB induction and retain site-specificity or a wild type Cas9 to integrate sequences through HDR-independent methods of sequence integration. Bhatt and Chalmers [86] described a fusion of dCas9 to the human *mariner* transposon *HsMar1* and tested its ability to integrate 4.5 kb of sequence containing a kanamycin resistance gene and *lacZα* gene into a donor plasmid. The dCas9-sgRNA-*HsMar1* construct was able to reach target efficiencies of greater than 50% whereas transformation of plasmids containing either dCas9-*HsMar1* or *HsMar1* alone into *E. coli* cells resulted in significantly lower targeting efficiencies ranging from 0 to 10% depending on target plasmid size [86]. One of these complexes reported by Ma et al. [87] relies on a homology-independent targeted integration (HITI) that cleaves donor DNA without HAs in both dividing and non-dividing cells. Ma et al.’s construct consisted of a Sleeping Beauty transposase DNA binding domain (N57) with Cas9 and a sgRNA targeting the *AAVSI* site in MCF7 human breast cancer cells. Using a plasmid DNA donor with an EGFP coding sequence, the Cas9-N57 construct increased integration of a 12-kb fragment twofold compared to a wild-type Cas9 [87]. This method allows for site-specific Cas9-sgRNA recruitment of a Cas9-N57 fusion construct that can integrate a gene of interest within donor DNA containing the corresponding binding sequence.

## Conclusions

Techniques that inhibit NHEJ or promote HDR to increase knock-in rates, such as those tested in mammalian systems, can provide important lessons for vector biologists seeking more efficient rates of transgene integration (Table 1). Though some factors are not conserved between mammalian and mosquito DSB repair pathways, many of the methods used to enhance HDR in mammals act through conserved mechanisms (i.e. by inhibiting competing pathways like NHEJ, or promoting important HDR processes such as end resection or strand invasion). While the individual strategies described herein each resulted in increases in homology-based repair, we emphasize that synergy between two or more approaches can further increase HDR-based sequence integration in the appropriate context. For example, it may be possible to combine the efficiency of modified nuclease systems with that of cell cycle-synchronization chemicals to further promote HDR in cell culture. In vivo, it may be possible to increase rates of transgene integration during embryo microinjections by combining modified nuclease systems with each other and/or in conjunction with small molecules that recruit pro-HDR factors. It is also important to note that there are other factors that can influence HDR efficiency, such as homology arm length, donor DNA type, and donor size; all of these aspects should be considered together when planning gene editing experiments. We hope that by reviewing DSB repair and how recent advancements in vertebrate genome editing may apply to mosquito systems, vector biologists can expand their molecular toolkit and advance the field of arbovirus control.

**Table 1:** Modified Cas9 approaches to increasing the frequency of homology-dependent repair outcomes

Approach	Rationale	Reported effect	References
Cas9-i53 (hypothetical)	Expression of i53 alone strongly suppressed the recruitment and function of 53BP1 at DSBs	Cas9-i53 fusion has not been reported, however expression of i53 in cell lines induces BRCA1 accumulation at DSB sites similar to 53BP1 knockout cells	Canny et al. [28]
Cas9-DN1S	DN1S inhibits the recruitment of key NHEJ proteins such as RIF1	two- to threefold increase in HDR and three- to fourfold reduction in NHEJ	Jayavaradhan et al. [24]
Cas9-yRAD52	yRAD52 can stimulate strand invasion and homologous recombination at a DSB site	Threefold HDR increase in human cells Threefold HDR increase in chicken cells	Shao et al. [44], Wang et al. [45]
Cas9-HE (CtIP)	CtIP assists in end-resection and promotes HDR	Twofold HDR increase in human cell lines, human iPSCs, and rat zygotes	Charpentier et al. [49]
Cas9-UL12	UL12 recruits endogenous MRN complex which resects DSBs and promotes HDR	Twofold HDR increase in HEK293 cells	Reuven et al. [51]
miCas9 (cas9-Brex27)	Brex27 recruits RAD51, a pro-HDR protein that searches for homologous sequences and facilitates D-loop formation at DSB sites	Up to threefold HDR increase depending on donor size in human cell lines	Ma et al. [56]
REDIT	Exploits bacteriophage SSAPs to insert kilobases of sequence at DSB sites	Threefold increase in HDR in human AECs and iPSCs	Wang et al. [61]
Cas9-RecA	RecA is able to promote single-strand annealing pathway at DSBs in between repetitive sequences	2.5-fold increase in SSA in HEK293T cells	Lin et al. [66]
Cas9-hGem	Restricts Cas9 presence and activity to late S and G2 when HDR occurs most frequently	1.87-fold HDR increase in HEK293T cells	Gutschner et al. [79]
Cas9-PCV	Ensures proximity of donor DNA at DSB site through PCV-ssDNA covalent bonding	Up to 30-fold increase in HDR correcting frameshift mutations with short ssDNA donor	Aird et al. [84]

## Data Availability

Not applicable.
